# Learning from negative feedback in patients with major depressive disorder is attenuated by SSRI antidepressants

**DOI:** 10.3389/fnint.2013.00067

**Published:** 2013-09-23

**Authors:** Mohammad M. Herzallah, Ahmed A. Moustafa, Joman Y. Natsheh, Salam M. Abdellatif, Mohamad B. Taha, Yasin I. Tayem, Mahmud A. Sehwail, Ivona Amleh, Georgios Petrides, Catherine E. Myers, Mark A. Gluck

**Affiliations:** ^1^Al-Quds Cognitive Neuroscience Lab, Faculty of Medicine, Al-Quds UniversityAbu Dis, Palestinian Territories; ^2^Center for Molecular and Behavioral Neuroscience, Rutgers UniversityNewark, NJ, USA; ^3^Marcs Institute for Brain and Behaviour, University of Western SydneySydney, NSW, Australia; ^4^School of Social Sciences and Psychology, University of Western SydneySydney, NSW, Australia; ^5^Hofstra North Shore-LIJ School of Medicine, The Zucker Hillside Hospital North Shore-LIJ Health SystemGlen Oaks, NY, USA; ^6^Department of Veterans Affairs, New Jersey Health Care SystemEast Orange, NJ, USA; ^7^Department of Neurology and Neurosciences, New Jersey Medical School/University of Medicine and Dentistry of New JerseyNewark, NJ, USA; ^8^Department of Psychology, Rutgers UniversityNewark, NJ, USA

**Keywords:** major depressive disorder, selective serotonin reuptake inhibitor, basal ganglia, reward, punishment

## Abstract

One barrier to interpreting past studies of cognition and major depressive disorder (MDD) has been the failure in many studies to adequately dissociate the effects of MDD from the potential cognitive side effects of selective serotonin reuptake inhibitors (SSRIs) use. To better understand how remediation of depressive symptoms affects cognitive function in MDD, we evaluated three groups of subjects: medication-naïve patients with MDD, medicated patients with MDD receiving the SSRI paroxetine, and healthy control (HC) subjects. All were administered a category-learning task that allows for dissociation between learning from positive feedback (reward) vs. learning from negative feedback (punishment). Healthy subjects learned significantly better from positive feedback than medication-naïve and medicated MDD groups, whose learning accuracy did not differ significantly. In contrast, medicated patients with MDD learned significantly less from negative feedback than medication-naïve patients with MDD and healthy subjects, whose learning accuracy was comparable. A comparison of subject’s relative sensitivity to positive vs. negative feedback showed that both the medicated MDD and HC groups conform to Kahneman and Tversky’s (1979) Prospect Theory, which expects losses (negative feedback) to loom psychologically slightly larger than gains (positive feedback). However, medicated MDD and HC profiles are not similar, which indicates that the state of medicated MDD is not “normal” when compared to HC, but rather balanced with less learning from both positive and negative feedback. On the other hand, medication-naïve patients with MDD violate Prospect Theory by having significantly exaggerated learning from negative feedback. This suggests that SSRI antidepressants impair learning from negative feedback, while having negligible effect on learning from positive feedback. Overall, these findings shed light on the importance of dissociating the cognitive consequences of MDD from those of SSRI treatment, and from cognitive evaluation of MDD subjects in a medication-naïve state before the administration of antidepressants. Future research is needed to correlate the mood-elevating effects and the cognitive balance between reward- and punishment-based learning related to SSRIs.

## INTRODUCTION

Major depressive disorder (MDD) is debilitating psychiatric disease, characterized by persistent low mood and significant loss of pleasure (Belmaker and Agam, 2008). MDD has been associated with various cognitive deficits, including alterations to learning from positive feedback (reward) and negative feedback (punishment; Eshel and Roiser, 2010). Behavioral studies suggest that patients with MDD show hypersensitive responses to punishment (Beats et al., 1996; Elliott et al., 1996, 1997), while being hyposensitive to reward (Henriques et al., 1994; McFarland and Klein, 2009; Robinson et al., 2012a). These findings fit with psychological theories of MDD, which argue that patients with MDD manifest abnormally negative attitudes and thoughts (Bower, 1981), while being unable to modulate their behavioral responses when presented with positive reinforcement, which results in misconception of environmental information to confirm these biases (Gotlib and Joormann, 2010; Roiser and Sahakian, 2013). Such cognitive biases relate to the underlying neural circuits that are affected by MDD, namely the basal ganglia and the limbic system (Sheline et al., 2001; Nutt, 2006; Dunlop and Nemeroff, 2007). Accordingly, we can draw two major conclusions from the literature on MDD patients’ ability to process information in the context of positive and negative feedback. The first is that patients with MDD show exaggerated responses to negative feedback (Beats et al., 1996; Elliott et al., 1996, 1997), while the second is that MDD patients show hyposensitive responses to positive feedback (Henriques et al., 1994; McFarland and Klein, 2009; Robinson et al., 2012a).

In addition to being implicated in the pathophysiology of MDD, the monoamines serotonin and dopamine have also been shown to be play major roles in reinforcement learning (Deakin, 1991; Dunlop and Nemeroff, 2007; Cools et al., 2011). Serotonin has been prominently associated with aversive processing as well as behavioral inhibition, where serotonin levels positively correlate with punishment-induced inhibition and aversive processing but not overall inhibition of motor responses to aversive outcomes (Deakin and Graeff, 1991; Crockett et al., 2009). Studies have shown that acute tryptophan depletion (a dietary technique used to reduce central serotonin concentrations) enhances reversal learning of aversive cues in healthy subjects (Cools et al., 2008), which mimics the feedback sensitivity bias in patients with MDD (Clark et al., 2009; Eshel and Roiser, 2010). Aside from being key for learning from positive feedback (Schultz et al., 1997), it has been suggested that dopaminergic dysregulation plays a central role in the cognitive correlates of MDD (Nutt, 2006; Dunlop and Nemeroff, 2007; Nutt et al., 2007). Imaging studies have shown that patients with MDD exhibit hyposensitive responses to reward alongside attenuated striatal response to presentation of reward (Henriques et al., 1994; McFarland and Klein, 2009; Robinson et al., 2012a). These reports highlight the low serotonergic and low dopaminergic state in MDD, which could represent the neurochemical basis for the observed cognitive biases in MDD (Cools et al., 2011).

A substantial proportion of patients with MDD respond to pharmacological treatment with antidepressants, including selective serotonin reuptake inhibitors (SSRIs; Carvalho et al., 2007), which are thought to achieve their therapeutic effect, primarily, by modifying synaptic availability of monoamines, namely serotonin, dopamine, and norepinephrine (Malberg and Schechter, 2005). Recent studies argue that SSRI administration in MDD results in normalization of activity in the prefrontal cortex (PFC) and amygdala (Di Simplicio et al., 2012; Godlewska et al., 2012), normalization of the functional connectivity between PFC and both hippocampus and amygdala (McCabe et al., 2011), and enhancement of reward learning and striatal activity (Stoy et al., 2011). On the other hand, reports suggest that the administration of SSRIs diminishes the processing of both reward and punishment stimuli in healthy subjects (McCabe et al., 2010), but diminishes learning from punishment stimuli and enhances learning from reward stimuli in rats (Bari et al., 2010). Accordingly, there is evidence that SSRI administration normalizes brain activity in key regions for learning from positive and negative feedback, and enhances learning from positive feedback. Unfortunately, relatively little is known about how the remediation of psychiatric symptoms by SSRIs impacts the balance between learning from reward and punishment in MDD.

In this study, our main aim was to investigate the effect of remediation of depressive symptoms by SSRI administration on the balance between learning from positive and negative feedback in MDD. We tested medication-naïve patients with MDD, SSRI-responder patients with MDD and matched healthy control (HC) subjects, on a computer-based learning task that uses a mix of positive-feedback and negative feedback (Bodi et al., 2009). To our knowledge, no previous studies attempted to dissociate the effects of MDD and SSRI on reward and punishment learning in the same study.

## MATERIALS AND METHODS

### PARTICIPANTS

We recruited and tested 13 medication-naïve patients with MDD, 18 SSRI-responding patients with MDD (MDD-T), and 22 HC subjects, from various psychiatric clinics, mental health care centers and primary health care centers throughout the West Bank, Palestinian Territories. All subjects were White, ranging from 20 to 60 years of age. Participants were group matched for age, gender, and years of education, as shown in **Table 1**. All subjects underwent screening evaluations that included a medical history and a physical examination. Psychiatric assessment was conducted using an unstructured interview with a psychiatrist using the DSM-IV-TR criteria for the diagnosis of MDD (melancholic subtype), and the Mini International Neuropsychiatric Interview (MINI; Amorim et al., 1998). We recruited medication-naïve patients with MDD after meeting the DSM-IV-TR criteria for MDD and completing the MINI structured clinical interview to confirm the diagnosis and absence of comorbidities. We tested medication-naïve patients with MDD immediately prior to their initiating treatment with SSRIs. All SSRI-treated patients with MDD received 10–30 mg of paroxetine per day (mean = 18.333, SD = 5.941) as part of their normal ongoing treatment. Inclusion criteria for HC subjects were absence of any psychiatric, neurological, or other disorders that might affect cognition. MDD-T patients’ average exposure to SSRIs was 12.833 (SD = 18.912) months. MDD-T patients’ response to SSRIs was assessed using subjective reports and scores on the Beck Depression Inventory II (BDI-II). Exclusion criteria for all subjects included psychotropic drug exposure, except for the SSRI paroxetine in the SSRI-treated MDD group; major medical or neurological illness; illicit drug use or alcohol abuse within the past year; lifetime history of alcohol or drug dependence; psychiatric disorders other than major depression (excepting comorbid anxiety symptoms); current pregnancy or breastfeeding. After receiving a complete description of the study, participants provided written informed consent as approved by both the Al-Quds University Ethics Committee and the Rutgers Institutional Review Board.

**Table 1:** Summary of demographic and neuropsychological results.

		Age	Education	MMSE	BDI-II	BAI	NS	HA	RD
HC	Mean	28.50	15.09	29.91	5.5	6.36	14.63	10.54	17.91
	SD	11.84	1.57	0.29	4.09	5.60	3.95	4.51	2.69
MDD	Mean	27.23	14.31	28.53	33.77	28.84	16.38	20.77	14.69
	SD	6.24	2.29	1.33	10.02	9.01	3.47	6.24	4.23
MDD-T	Mean	32.11	13.56	27.83	9.72	9.27	14.78	15.83	18.50
	SD	9.14	2.17	2.71	6.41	5.43	3.21	5.36	3.07

*HC, healthy controls; MDD, medication-naïve patients with MDD; MDD-T, SSRI-treated patients with MDD; MMSE, Mini-Mental Status Exam; BDI-II, Beck Depression Inventory II; BAI, Beck Anxiety Inventory; TPQ dimensions, Tridimensional Personality Questionnaire; HA, harm avoidance; RD, reward dependence, NS, novelty seeking.*

### PSYCHOMETRIC AND PSYCHOPATHOLOGY TEST BATTERY

All subjects completed the validated Arabic version (Herzallah et al., 2010, 2013) of a battery of psychometric and psychopathology test questionnaires: Mini-Mental Status Examination (MMSE; Folstein et al., 1975), BDI-II (Beck et al., 1996), and Beck Anxiety Inventory (BAI; Beck et al., 1988). Further, all subject completed the Tridimensional Personality Questionnaire (TPQ; Cloninger et al., 1991). All results are summarized in **Table 1**.

### COMPUTER-BASED COGNITIVE TASK

#### Reward and punishment learning

**FIGURE 1:**
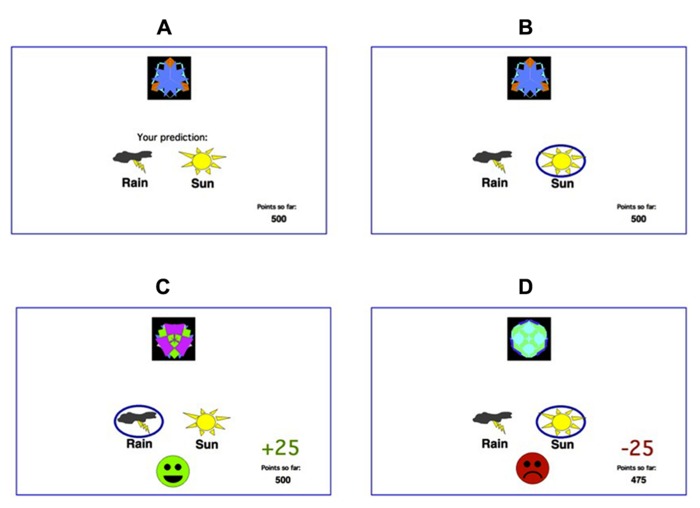
**The feedback-based deterministic classification task. (A)** On each trial, the participant saw one of eight stimuli and was asked whether this stimulus predicts rain or sun. **(B)** No feedback is given for incorrect answers in rewarding stimuli or correct answers in punishing stimuli. **(C)** For rewarding stimuli, correct responses get rewarded with visual feedback and 25 points winnings. **(D)** For punishing stimuli, incorrect responses get punished with visual feedback and the loss of 25 points.

Participants were administered a computer-based classification task (Bodi et al., 2009). On each trial, participants viewed one of eight images (**Figure 1**), and were asked to guess whether that stimulus predicts rainy weather (Rain, **Figure 1**) or sunny weather (Sun, **Figure 1**). For each participant, the eight images were randomly assigned to be stimuli S1–S8. On any given trial, stimuli S1, S3, S5, and S7 predicted Rain, while stimuli S2, S4, S6, and S8 predicted Sun. Stimuli S1–S4 were used in the reward-learning task. Four stimuli per valence were employed in order to balance category outcome frequencies, so that one stimulus in each task would be associated with each outcome. Thus, if the participant correctly guessed category membership on a trial with either of these stimuli, a reward of +25 points was received; if the participant guessed incorrectly, no feedback appeared. Stimuli S5–S8 were used in the punishment-learning task. Thus, if the participant guessed incorrectly on a trial with either of these stimuli, a punishment of –25 was received; correct guesses received no feedback.

The experiment was conducted on a Macintosh MacBook, programed in the SuperCard language. The participant was seated in a quiet testing room at a comfortable viewing distance from the screen. The keyboard was masked except for two keys, labeled “Sun” and “Rain” which the participant could use to enter responses. At the start of the experiment, the participant read the following instructions: “Welcome to the Fortuneteller School! You will be trained as a fortune teller to predict the weather. You learn to do this by using cards that either predict rain or sun. Your goal is to learn which cards predict rain and which cards predict sun.” The practice phase then walked the participant through an example of a correct and an incorrect response to a sample trial in the reward-learning task and an example of a correct and response to a sample trial in the punishment-learning task. These examples used images other than those assigned to S1–S8. The participant saw a practice image, with a prompt to choose “Sun” or “Rain,” and a running tally of points at the lower right corner of the screen. The tally was initialized to 500 points at the start of practice. The participant was first instructed to press the “Sun” key, which resulted in a reward of +25 and updated point tally and then the “Rain” key, which resulted in no feedback. The participant then saw a second practice figure and was instructed first to press the “Rain” key, which resulted in a reward of –25 and updated point tally and then the “Sun” key, which resulted in no feedback. After these two practice trials, a summary of instructions appeared: “So... for some pictures, if you guess CORRECTLY, you WIN points (but, if you guess incorrectly, you win nothing). For other pictures, if you guess INCORRECTLY, you LOSE points (but, if you guess correctly, you lose nothing). Your job is to win all the points you can and lose as few as you can. Press the mouse button to begin the experiment”. From here, the experiment began. In each trial, the participant saw one of the eight stimuli (S1–S8) and was prompted to guess whether it was a “Sun” or a “Rain.” On trials in the reward-learning task (with stimuli S1–S4), correct answers were rewarded with positive feedback and a gain of 25 points; incorrect answers received no feedback. On trials in the punishment-learning task (with stimuli S5–S8), incorrect answers were punished with negative feedback and a loss of 25 points; correct answers received no feedback. The task contained 160 trials, distributed over four blocks of 40 trials. Within a block, trial order was randomized. Trials were separated by a 1 s interval, during which time the screen was blank. Within each block, each stimulus appeared five times. Thus, training on the reward-learning task (S1–S4) and punishment-learning task (S5–S8) were intermixed. The no-feedback outcome, when it arrived, was ambiguous, as it could signal lack of reward (if received during a trial with S1–S4) or lack of punishment (if received during a trial with S5–S8).

### STATISTICAL ANALYSIS

The normality of data distribution was checked using Kolmogorov–Smirnov tests. All data were normally distributed (*p* > 0.1). We used mixed-design three-way ANCOVA followed by mixed-design two-way ANOVA and one-way ANOVA *post hoc* tests, Tukey’s honestly significant difference (HSD) *post hoc* tests and Bonferroni *post hoc* tests. The level of significance was set at α = 0.05.

## RESULTS

### BEHAVIORAL RESULTS

We used one-sample *t*-test on the percentage of correct responses in the fourth block of learning in both reward and punishment to ensure that subjects learned significantly better than chance in different groups. In reward learning, MDD-T and HC learned significantly better than chance, with Bonferroni correction adjusted α = 0.017 to protect the level of significance [MDD-T: *t*(17) = 3.264, *p* = 0.005; HC: *t*(21) = 9.997, *p* < 0.001], while MDD did not [*t*(12) = 0.925, *p* = 0.373]. In punishment learning, all groups learned significantly better than chance, with Bonferroni correction adjusted α = 0.017 to protect the level of significance [MDD: *t*(12) = 7.704, *p* < 0.001; MDD-T: *t*(17) = 3.394, *p* = 0.003; HC: *t*(11) = 13.231, *p* < 0.001].

**FIGURE 2:**
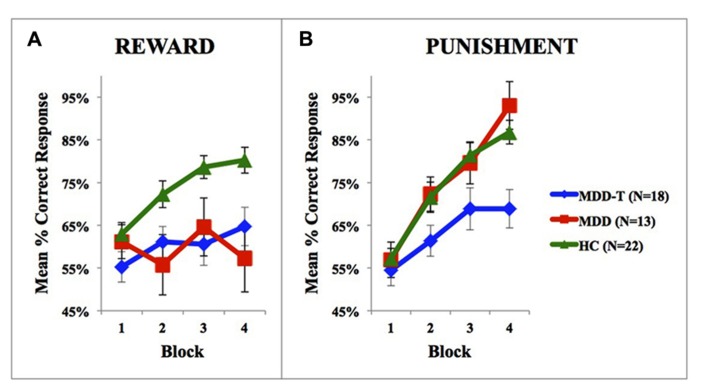
**Performance on the reward and punishment learning task; (A)** The mean number of correct responses in the four phases for the reward stimuli (±SEM). **(B)** The mean number of correct responses in the four phases for the punishment stimuli (±SEM). MDD is medication naïve, MDD-T is on medication MDD patients, and HC is healthy controls.

Using mixed-design three-way ANCOVA, we analyzed the data obtained from the cognitive task with group as the between-subject variable, learning block, and feedback type as within-subject variables, BDI-II scores as a covariate, and the percentage of correct responses on reward and punishment as the dependent variables. There was a significant effect of group [*F*(2,51) = 9.433, *p* < 0.001, η^2^ = 0.270] and block [*F*(3,153) = 11.880, *p* < 0.001, η^2^ = 0.189] as illustrated in **Figure 2**. However, there was no significant effect of feedback type [*F*(1,51) = 1.337, *p* = 0.253]. We conducted two *post hoc* mixed-design two-way ANOVAs, with group as the between-subject variable, learning block as within-subject variable, the percentage of correct responses on reward as the dependent variable in one of the ANOVAs and the percentage of correct responses on punishment in the other, and Bonferroni correction adjusted α = 0.025 to protect the level of significance. The reward *post hoc* revealed a significant effect of group [*F*(2,50) = 5.094, *p* = 0.010, η^2^ = 0.169] and block [*F*(3,150) = 6.000, *p* = 0.001, η^2^ = 0.107] along with an interaction between group and block [*F*(6,150) = 3.098, *p* = 0.007, η^2^ = 0.110]. We used four *post hoc* one-way ANOVAs to explore the significant interaction between group and block, with group as the between-subject variable, and the percentage of correct responses on a each one of the four reward learning block was the within-subject variable, with a Bonferroni correction adjusted α = 0.0125 to protect the level of significance. One-way ANOVA and Tukey’s HSD results are summarized in **Table 2**. The punishment *post hoc* two-way ANOVA showed a significant effect of group [*F*(2,50) = 4.512, *p* = 0.016, η^2^ = 0.153] and block [*F*(3,150) = 45.644, *p* < 0.001, *η*^2^ = 0.477], but no interaction between group and block [*F*(6,150) = 2.426, *p* = 0.029]. Tukey’s HSD *post hoc* test revealed a significant difference between MDD-T and both MDD and HC (*p* < 0.05), but not between MDD and HC.

**Table 2:** Summary of the *post hoc* one-way ANOVA and Tukey’s HSD *post hoc* results to explore the significant interaction between group and block in reward learning, with group as the between-subject variable, and the percentage of correct responses on a each one of the four reward learning block was the within-subject variable, with a Bonferroni correction adjusted α = 0.0125 to protect the level of significance.

Statistical test	Within-subject variable	Between-subject variable	df-1	df-2	*F*	*p*	η^2^
One-way ANOVA	Block 1 reward	Group (MDD, MDD-T, HC)	2	50	1.571	0.218	–
One-way ANOVA	Block 2 reward	Group (MDD, MDD-T, HC)	2	50	3.862	0.28	–
One-way ANOVA	Block 3 reward	Group (MDD, MDD-T, HC)	2	50	4.973	0.011*	0.166
Tukey’s HSD		HC vs. MDD-T	–	–	–	0.04*	–
		HC vs. MDD	–	–	–	0.097	–
		MDD vs. MDD-T	–	–	–	0.827	–
One-way ANOVA	Block 4 reward	Group (MDD, MDD-T, HC)	2	50	6.038	0.004*	0.194
Tukey’s HSD		HC vs. MDD	–	–	–	0.006*	–
		HC vs. MDD-T	–	–	–	0.049*	–
		MDD vs. MDD-T	–	–	–	0.572	–

HC, healthy controls; MDD, medication-naïve patients with MDD; MDD-T, SSRI-treated patients with MDD. The symbol “*” marks significant results.

**FIGURE 3:**
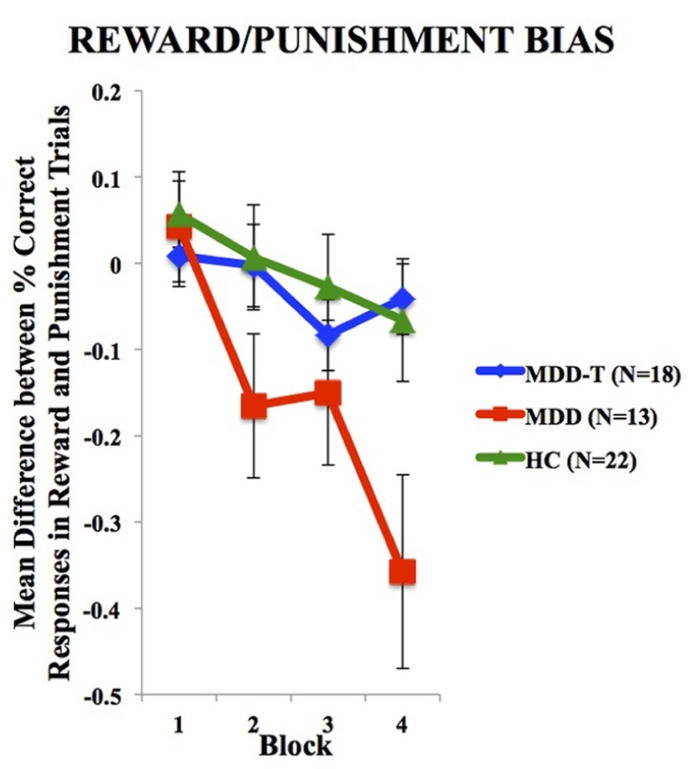
**Mean difference between percentage correct responses in reward and punishment trials per block (±SEM)**. MDD is medication naïve, MDD-T is on medication, and HC is healthy controls.

To investigate the balance between reward and punishment learning, we subtracted punishment learning accuracy in a particular block from that of reward in the same block. Two-way ANOVA, with group as the between-subject variable, block of learning as the within subject variable, and the mean difference between percentage correct responses in reward and punishment trials as the dependent variable, revealed a significant effect of block [*F*(3,150) = 11.147, *p* < 0.001, η^2^ = 0.182] and an interaction between block and group [*F*(6,150) = 3.145, *p* = 0.006, η^2^ = 0.112], but no significant effect of group [*F*(2,50) = 2.486, *p* = 0.094], as illustrated in **Figure 3**. We used four *post hoc* one-way ANOVA and Tukey’s HSD *post hoc* analyses on each block of mean difference between percentage correct responses in reward and punishment trials to investigate the interaction between block and group, with group as the between subject variable and the mean difference between percentage correct responses in reward and punishment trials as the dependent variable. ANOVA and Tukey’s HSD results are reported in **Table 3**.

**Table 3:** Summary of the *post hoc* one-way ANOVA and Tukey’s HSD *post hoc* analyses on each block of mean difference between percentage correct responses in reward and punishment trials to investigate the interaction between block and group, with group as the between subject variable and the mean difference between percentage correct responses in reward and punishment trials as the dependent variable.

Statistical test	Within-subject variable	Between-subject variable	df-1	df-2	*F*	*p*	η^2^
One-way ANOVA	Block 1 difference	Group (MDD, MDD-T, HC)	2	50	0.358	0.701	–
One-way ANOVA	Block 2 difference	Group (MDD, MDD-T, HC)	2	50	2.121	0.131	–
One-way ANOVA	Block 3 difference	Group (MDD, MDD-T, HC)	2	50	1.035	0.363	–
One-way ANOVA	Block 4 difference	Group (MDD, MDD-T, HC)	2	50	5.251	0.009*	0.173
Tukey’s HSD		HC vs. MDD	–	–	–	0.017*	–
		HC vs. MDD-T	–	–	–	0.963	–
		MDD vs. MDD-T	–	–	–	0.013*	–

HC, healthy controls; MDD, medication-naïve patients with MDD; MDD-T, SSRI-treated patients with MDD. The symbol “*” marks significant results.

### PSYCHOMETRIC RESULTS

There was no significant effect of group on age, education, MMSE score, or the novelty seeking subsection of the TPQ, with Bonferroni correction adjusted α = 0.006 to protect the level of significance (*p* > 0.006). However, there was a significant difference between groups in BDI-II scores [*F*(2,50) = 77.576, *p* < 0.001, η^2^ = 0.756, Tukey’s HSD *post hoc*: significant difference between MDD and both MDD-T and HCs], BAI scores [*F*(2,50) = 52.444, *p* < 0.001, η^2^ = 0.677, Tukey’s HSD *post hoc*: significant difference between MDD and both MDD-T and HCs], harm avoidance subsection of the TPQ [*F*(2,50) = 15.903, *p* < 0.001, η^2^ = 0.389, Tukey’s HSD *post hoc*: significant difference between HC and both MDD and MDD-T, and between MDD-T and HC], and reward dependence subsection of the TPQ [*F*(2,50) = 5.808, *p* = 0.005, η^2^ = 0.189, Tukey’s HSD *post hoc*: significant difference between HC and both MDD and MDD-T].

## DISCUSSION

We have three main findings. First, SSRI-treated patients with MDD were less sensitive to negative feedback (punishment) than either medication-naïve patients with MDD or HC subjects, based on their accuracy in the cognitive task. Second, both medication-naïve and SSRI-treated patients with MDD were less sensitive to positive-feedback than HC subjects. Third, a comparison of subjects’ learning from positive vs. negative feedback, showed that both the HC and MDD groups conform to Kahneman and Tversky’s (1979) Prospect Theory, which expects losses (negative feedback) to loom psychologically larger than gains (positive feedback; Kahneman and Tversky, 1979). In contrast the MDD patients violate Prospect Theory by being significantly more biased toward negative.

### BEHAVIORAL AND NEURAL CORRELATES OF MDD

Abnormal exaggerated reactions to negative events and overlooking positive events are considered central features of MDD (Beats et al., 1996; Elliott et al., 1996). These abnormal responses to positive and negative feedback represent an important link between emotional and cognitive disturbances in MDD (Wright and Beck, 1983; Elliott et al., 1997), showing an increased elaboration of negative information (Gotlib and Joormann, 2010), while ignoring positive information. As explained by the cognitive theory of depression (Clark and Beck, 2010); depressed people tend to demonstrate selective attention to negative information; magnifying the importance and meaning placed on negative events (Beck, 1979; Bower, 1981). Our results show that medication-naïve patients with MDD learn from punishment as efficiently as HC subjects, but fail to learn from reward feedback. However, the task design we use in the current study is not the most ideal approach to delineate higher-than-normal learning from punishment learning in MDD due to a possible ceiling effect (**Figure 2B**). Further research is needed in this domain to further investigate the differential sensitivity to negative feedback in MDD as compared to healthy subjects, and properly correlate cognitive measures with symptom distribution and severity in patients with MDD.

Patients with MDD’s strong biases toward negative stimuli and away from positive ones highlights the role of serotonin in the processing of affective stimuli and inhibitory control of behavior and adaptation of the animals to aversive events (Graeff et al., 1996), and underpin the attentional bias in MDD toward negative feedback (Mogg et al., 1995; Harmer et al., 2009). Lowering brain serotonin level by acute tryptophan depletion (serotonin precursor) in healthy volunteers results in increased sensitivity to punishment and negative feedback without affecting reward (Cools et al., 2008; Robinson et al., 2012b). These alterations in the reward and punishment processing implicate a neural circuit that is composed of brain regions strongly innervated by serotonin, namely, the medial PFC and the ventral striatum (Clark et al., 2009).

Recent imaging studies argue that patients with MDD manifest cognitive and neurochemical dysfunction directly related to the nigrostriatal dopaminergic system (Dunlop and Nemeroff, 2007; Walter et al., 2007; Robinson et al., 2012a). On the other hand, previous research has shown that the basal ganglia dopaminergic system is vital for learning to predict rewarding outcomes (Schultz et al., 1997; Haber and Knutson, 2010). In a previous study using a reward-punishment learning task (similar to the task we used in this paper), we demonstrated that medication-naïve patients with Parkinson’s disease learned very well from punishment but were impaired on reward learning (Bodi et al., 2009). Our findings indicate that medication-naïve patients with MDD show similar cognitive profile to de novo patients with Parkinson’s (Bodi et al., 2009). Both disorders were shown to suppress learning from reward (Henriques et al., 1994; Bodi et al., 2009; McFarland and Klein, 2009; Robinson et al., 2012a), without altering learning from punishment (Beats et al., 1996; Elliott et al., 1996, 1997; Bodi et al., 2009). This observation might be attributed to the effect of both disorders on the striatal dopamine (Kish et al., 1988; Walter et al., 2007). Further, there is a very high level of comorbidity between MDD and Parkinson’s disease (Cummings, 1992; Schuurman et al., 2002; Leentjens et al., 2003; Veiga et al., 2009). However, it is not clear whether this overlap between the two disorders is a consequence of dopaminergic dysfunction alone, or it is a mixture monaminergic effects (Kitaichi et al., 2010; Delaville et al., 2012). In addition, our findings suggest that SSRI-treated patients with MDD learn significantly less than HC subjects from positive-feedback, similar to medication-naïve patients with MDD. Future studies ought to compare the cognitive correlates of SSRI administration in MDD and depression in Parkinson’s disease.

Increasing the central level of serotonin by administration of SSRIs counteracts MDD-related negative biases in aversive learning paradigms in animals (Bari et al., 2010) as well as emotional learning paradigms in humans (Harmer et al., 2009; McCabe et al., 2010). Various studies show that the administration of SSRIs normalizes the BOLD response in the dorsomedial PFC and across the functional connection between PFC and both hippocampus and amygdala (McCabe et al., 2011). Hence, it has been proposed that SSRIs may ameliorate MDD symptoms by inhibiting processing of negative feedback (Boureau and Dayan, 2011; Cools et al., 2011). In agreement with these results, we found here that SSRI-treated patients with MDD are less sensitive to negative feedback as compared to both medication-naïve patients with MDD as well as HC subjects.

In Watts et al. (2012), daily administration of SSRIs caused normal rats to slowly begin to lose selectivity in their box-checking behavior for food reward; they soon began to check more unbaited boxes. If SSRI administration reduces salience of punishment, it may be that the Watts et al.’s (2012) behavioral outcome is not due to lack of consolidation or reconsolidation of which boxes were baited or unbaited, as the authors chose to interpret their findings, but rather resulted from a lack of motivation to discriminate the rewarded vs. unrewarded boxes since the slight negative drawback (waste of time and effort) of checking an unbaited box was no longer worth the cognitive effort of discrimination. This could support either a learning deficit with MDD treatment or a loss of the power of negative motivation, or both. However, it also remains possible that change in the MDD-T performance in our study is due to an *a priori* learning impairment caused by the MDD treatment, or the effects of recovery from MDD. All groups did seem to learn the positive reward stimuli, but none of them learned it well, whereas the MDD and HC groups learned from punishment quite well indeed while the MDD-T group poor learning from punishment compares to their poor learning from reward.

Driven by the SSRI-related suppression of punishment learning, we found that SSRI-treated patients with MDD expressed balanced reward-punishment learning bias similar to HC subjects. This balance can be the underlying mechanism for SSRI-induced restoration of mood in patients with MDD. It is worth noting, however, that SSRI-treated MDD and HC profiles are not similar, which indicates that the state of SSRI-treated MDD is not “normal” (when compared to HC), but rather balanced with less learning from both positive and negative feedback. The negative values in this difference computation for the HC and MDD-T groups indicated a biased sensitivity to learn slightly more quickly from negative feedback (punishment) than positive feedback (reward) as expected by Kahneman and Tversky’s (1979) Prospect Theory, which expects that losses from negative feedback should loom larger than gains from positive feedback. Only the MDD group failed to conform to the Prospect Theory with significantly exaggerated bias toward negative feedback.

### LIMITATIONS AND FUTURE DIRECTIONS

An important limitation of the current study is that the different severity of depressive symptoms in SSRI-treated vs. medication-naïve patients might have contributed to the difference between the groups. We did not have access to SSRI-treated patients’ BDI-II scores before they were placed on the SSRI regimen. Therefore, it is impossible to conclude that the observed behavioral effects originate from the medication alone. However, we added BDI-II scores as a covariate in our main analysis, and matched the different groups on a number of psychometric measures.

Another major limitation to our study is the between-subject design, where the medication-naïve and the SSRI-treated patients with MDD are different individuals. Given the heterogeneity of MDD, and how various subtypes of MDD differ with regards to cognitive function, the current result might be confounded by between-subject variability originating from factors other than MDD and SSRI administration. Further, given that we recruited SSRI-responders, it is not expected that the selected medication-naïve patients with MDD will turn out to be responders once they started SSRI monotherapy, which limits the comparability of the groups and represents a major limitation of the current study. We did, of course, try to control for that in the current study by recruiting melancholic patients with MDD only, and by matching the two groups on various psychometric and demographic measures as described earlier. However, future work ought to address this issue by examining the same patients with MDD on and off medication. Another limitation of the current study is the low number of recruited subjects. However, given that the focus of the current study is cognitive function assessment, all *a priori* power analyses indicated the need for 14 subjects per group to achieve power levels higher than 90%, which confirms the sufficiency of the number of subjects in the analysis of our primary cognitive results. Future studies, however, should address these limitations and better control for possible confounding variables.

## Conflict of Interest Statement

The authors declare that the research was conducted in the absence of any commercial or financial relationships that could be construed as a potential conflict of interest.
